# AutoCNV: a semiautomatic CNV interpretation system based on the 2019 ACMG/ClinGen Technical Standards for CNVs

**DOI:** 10.1186/s12864-021-08011-4

**Published:** 2021-10-06

**Authors:** Chunna Fan, Zhonghua Wang, Yan Sun, Jun Sun, Xi Liu, Licheng Kang, Yingshuo Xu, Manqiu Yang, Wentao Dai, Lijie Song, Xiaoming Wei, Jiale Xiang, Hui Huang, Meizhen Zhou, Fanwei Zeng, Lin Huang, Zhengfeng Xu, Zhiyu Peng

**Affiliations:** 1 College of Life Sciences, University of Chinese Academy of Sciences, 100049 Beijing, China; 2 Tianjin Medical Laboratory, BGI-Tianjin, BGI-Shenzhen, 300308 Tianjin, China; 3 Binhai Genomics Institute, BGI-Tianjin, BGI-Shenzhen, 300308 Tianjin, China; 4 BGI Genomics, BGI-Shenzhen, 518083 Shenzhen, China; 5 BGI-Wuhan Clinical Laboratories, BGI-Shenzhen, 490079 Wuhan, China; 6 Department of Biology, Faculty of Science, University of Copenhagen, DK-2200 Copenhagen, Denmark; 7 State Key Laboratory of Reproductive Medicine, Department of Prenatal Diagnosis, Women’s Hospital of Nanjing Medical University, Nanjing Maternity and Child Health Care Hospital, 210004 Nanjing, China

**Keywords:** AutoCNV, CNV interpretation, Scoring, CNV classification

## Abstract

**Background:**

The American College of Medical Genetics and Genomics (ACMG) and the Clinical Genome Resource (ClinGen) presented technical standards for interpretation and reporting of constitutional copy-number variants in 2019 (the standards). Although ClinGen developed a web-based CNV classification calculator based on scoring metrics, it can only track and tally points that have been assigned based on observed evidence. Here, we developed AutoCNV (a semiautomatic automated CNV interpretation system) based on the standards, which can automatically generate predictions on 18 and 16 criteria for copy number loss and gain, respectively.

**Results:**

We assessed the performance of AutoCNV using 72 CNVs evaluated by external independent reviewers and 20 illustrative case examples. Using AutoCNV, it showed that 100 % (72/72) and 95 % (19/20) of CNVs were consistent with the reviewers’ and ClinGen-verified classifications, respectively. AutoCNV only required an average of less than 5 milliseconds to obtain the result for one CNV with automated scoring. We also applied AutoCNV for the interpretation of CNVs from the ClinVar database and the dbVar database. We also developed a web-based version of AutoCNV (wAutoCNV).

**Conclusions:**

AutoCNV may serve to assist users in conducting in-depth CNV interpretation, to accelerate and facilitate the interpretation process of CNVs and to improve the consistency and reliability of CNV interpretation.

**Supplementary Information:**

The online version contains supplementary material available at 10.1186/s12864-021-08011-4.

## Background

As a major component of human genetic variation, copy number variants (CNVs) are often reported to be associated with human diseases, such as congenital malformations and intellectual disability, developmental delay, and autism spectrum disorder [1, 2]. Changes in gene dosage, gene fusion, gene disruption, or position effects caused by CNVs may lead to a wide variety of diseases in humans. To date, a number of methods have been developed for the detection of CNVs at the genome scale, including microarray-based methods^3^ and massively parallel sequencing (MPS) [3, 4]. The resolution of CNV detection strongly improved with the rapid development of MPS, progressing from the chromosome level down to the single gene/exon level. Despite the rapid advances in the methodology of CNV detection, the interpretation and understanding of CNVs is still lagging.

To facilitate the interpretation of CNVs, many databases have been developed, such as ClinGen [5], DECIPHER [6] and the Database of Genomic Variants (DGV) [7]. For the purpose of CNV dosage analysis, the International Standards for Cytogenomic Assays (ISCA) Consortium began to evaluate genomic regions and genes in 2011 [8]. Storing CNV information and the CNV-disease relationship of individuals affected with genetic diseases and normal individuals along with accumulating scientific literature is highly useful in facilitating the interpretation of CNVs. Despite the rapid development of databases and scientific literature for CNV analysis, the establishment of systematic guidelines for CNV interpretation is necessary to facilitate the process of CNV interpretation and to promote the consistency of interpretation.

To standardize CNV interpretation in clinical settings, the ACMG (the American College of Medical Genetics and Genomics) reported the recommended standards for CNV interpretation in 2011 [9]. This guideline primarily focused on CNVs generated using microarray-based technology [9]. With the continued widespread implementation of microarray-based and MPS technology, various types of CNVs have been identified and reported, which makes the interpretation of CNVs more complicated and challenging. To reduce discordance in the clinical classification of CNVs, the ACMG and ClinGen updated the guidelines (the 2019 ACMG/ClinGen Technical Standards for CNVs) for CNV interpretation in 2019 [1]. In the 2019 ACMG/ClinGen Technical Standards for CNVs (the standards), a quantitative, evidence-based scoring framework combining a total of 80 criteria was developed, and a five-tier classification system (pathogenic, likely pathogenic, uncertain significance, likely benign, and benign) was recommended for CNV interpretation [1].

Although a more comprehensive criteria and scoring system was provided by the ACMG and ClinGen, the successful application of these guidelines in the process of CNV interpretation remains challenging. First, a standardized scoring pipeline is required to ensure reproducibility. In this context, reproducibility means that the results generated by the same clinical scientist are consistent at different times. CNV interpretation is highly complicated and requires highly specific expertise in this domain. Without a standardized pipeline, the results of CNV interpretation might not be reproducible by the same clinical scientist due to human error. Second, a standardized scoring pipeline is required to reduce inconsistencies among different clinical scientists in the same lab or different labs. Without a standardized pipeline, inconsistency may be easily caused by the usage of different tools and databases in the CNV interpretation process. However, a standardized scoring pipeline is difficult to develop for many clinical scientists at present. Third, the complete fulfillment of the updated standards in the CNV interpretation process for clinical scientists remains complicated and time-consuming. For each standard, various tools and databases must be used. Using these tools, searching databases and gathering the results for a specific CNV is complicated and time-consuming. Some tools have been developed to overcome these challenges, such as ClassifyCNV [10] and AnnotSV [11]. However, ClassifyCNV is a command-line program, a web-based version of ClassifyCNV should be developed to provide a more user-friendly way for CNV interpretation, especially for clinical scientists. As for AnnotSV, no options of user adjustment can be made in the web-based version of AnnotSV. Some criteria still require the input of point values by users to generate final classification of the CNV according to case information from published studies, databases, internal lab data, or inheritance pattern/family history for the case being studied. Considering all these issues for CNV interpretation, a highly automated tool is urgently needed to combine all the databases and to provide a standardized pipeline for CNV interpretation.

In this study, we developed a tool, AutoCNV, to enable consistent and reliable CNV interpretation based on the various standards. AutoCNV included a total of 6 databases and implemented all 80 criteria for the annotation and interpretation of CNVs. We also tested the performance of AutoCNV using 72 CNVs evaluated by external independent reviewers [1], 20 illustrative case examples and data from the dbVar database [12]. This report presents a crucial first step in the establishment of a standardized workflow for CNV interpretation and paves the way for the clinical application of various CNV detection methods and for the integration of scientific investigations and clinical practice. AutoCNV is freely available at https://github.com/zhonghua-wang/autocnv.

## Results

### Features and functionality of AutoCNV

**Fig. 1:**
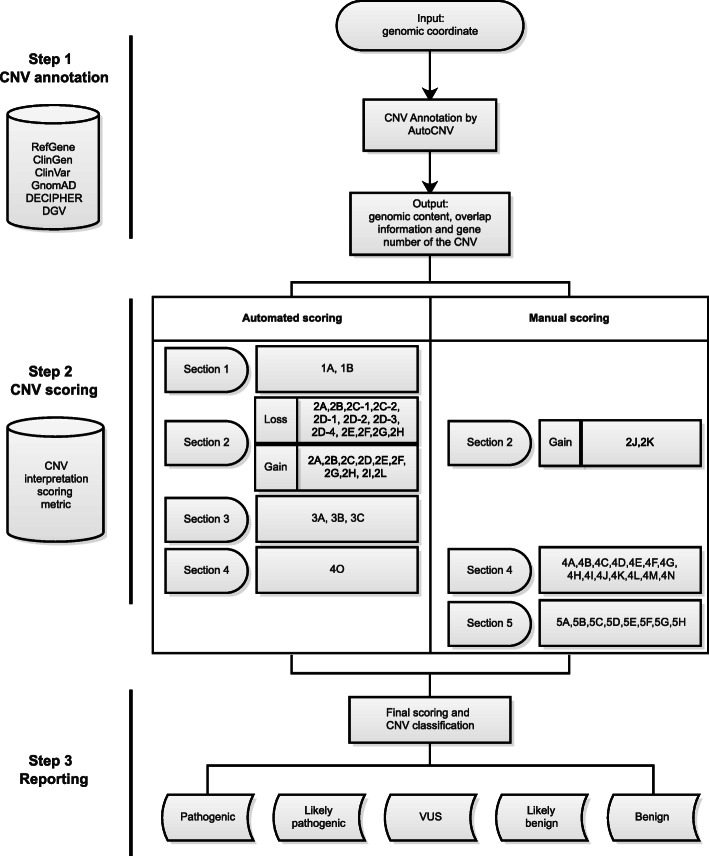
Workflow of the three steps of AutoCNV

There are 3 steps for AutoCNV: CNV annotation, CNV scoring, and reporting (Fig. 1). First, AutoCNV performs annotation for CNV interpretation according to user input. Second, AutoCNV implemented a scoring system according to Tables 1 and 2 in the standards [1]. Finally, a five-tier classification for both copy-number loss and copy-number gain (i.e., pathogenic, likely pathogenic, uncertain significance, likely benign, and benign) is generated. The CNV classification of AutoCNV is consistent with the CNV interpretation scoring framework reported in the standards (pathogenic: 0.99 or more points; likely pathogenic: 0.90 to 0.98 points; variant of uncertain significance: -0.89 to 0.89 points; likely benign: -0.90 to -0.98 points; benign: -0.99 or fewer points) [1].

### Workflow of AutoCNV

To illustrate the workflow of AutoCNV, we introduced a clinical case (Case 1) as an example. Case 1, a 35-year-old female, exhibited nephrotic syndrome (HP:0000100) and syncope (HP:0001279), and the WES result showed an approximate 1.5-Mb heterozygous copy number loss in chromosome 22 with coordinates of 18,761,827 − 20,307,561 (GRCh37/hg19). In this instance, we showed the scoring logic for the detected deletion of Case 1 as an example to illustrate the workflow of AutoCNV.

1. Automated scoring on each of the 18 criteria

Users can use the genomic coordinate of the CNV (chr22: 18,761,827 − 20,307,561) as input. According to the annotation result of the deletion, it spans protein-encoding genes, and a suggested point value of 0 (category 1 A) was first assigned by AutoCNV automatically. The deletion completely spans an established HI genomic region (nssv1184570, chr22: 18,912,231 − 20,287,208), and a suggested point value of 1 (category 2 A) was then assigned. The deletion contains a total of 32 protein-encoding genes, and a suggested point value of 0.45 (category 3B) was further added. In this section, a total of 1.45 suggested points were assigned to the deletion by automated scoring.

2) Manual review and adjustment of specific criteria

A score of > 0.99 was obtained, and the CNV classification of the deletion was made before manual adjustment; therefore, no further assessment was needed. In this part, no suggested point value was assigned to the deletion by manual scoring.

3) Final scoring and CNV classification to arrive at a final interpretation

A final point value of 1.45 was automatically generated by AutoCNV. A corresponding CNV classification of “pathogenic” was then assigned to the deletion.

### wAutoCNV: web version of AutoCNV

**Fig. 2:**
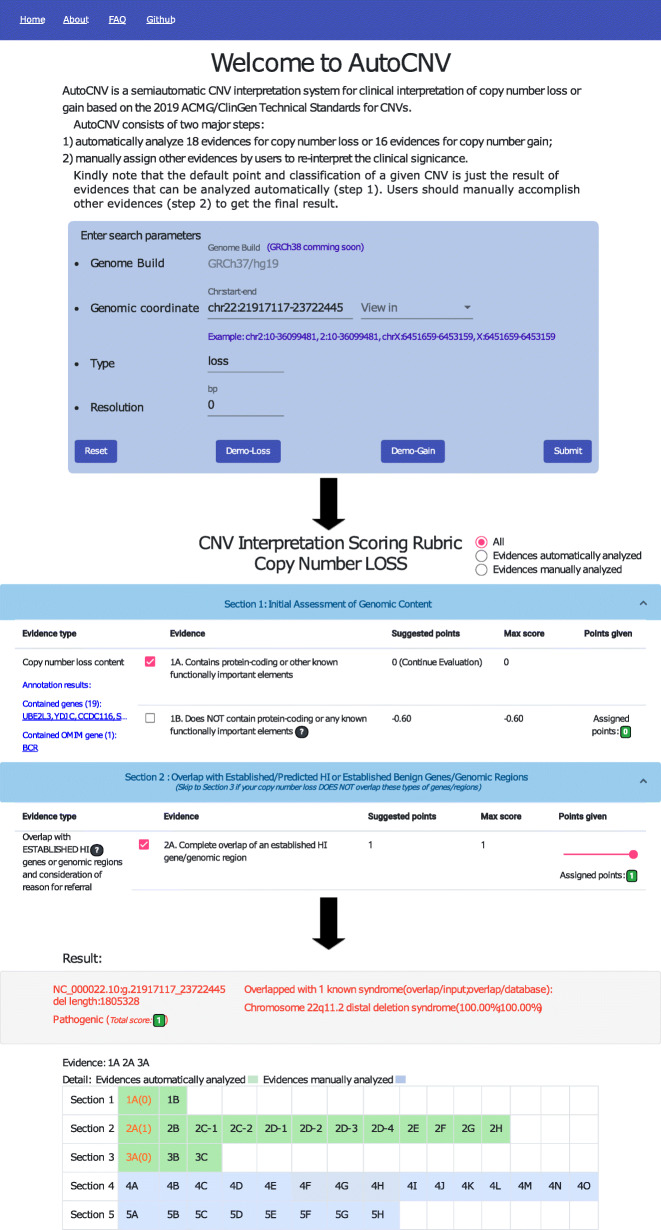
Illustration of wAutoCNV

AutoCNV is a command-line program written in Python. The source code is available on GitHub with a free license for noncommercial use (https://github.com/zhonghua-wang/autocnv). wAutoCNV (https://phoenix.bgi.com/autocnv/) is a web-based version of AutoCNV that can provide user-friendly CNV interpretation for clinical scientists. Clinical scientists can directly input CNVs into wAutoCNV by chromosomal coordinates (Fig. 2). wAutoCNV performs annotation analysis first according to the user input. Next, automated interpretation is performed. The wAutoCNV server provides detailed criteria and supportive evidence for the CNV, which can then be used for user-specific adjustments. After adjustment, wAutoCNV generates a final interpretation for the CNV. Clinical scientists can quickly search specific CNVs and reach a final classification.

In the standards, to allow user flexibility, a range is provided for most scoring categories. Taking category 2D-4 for copy number loss as an example, if there is additional evidence to suggest a detrimental effect on the protein, upgrading from the default score is suggested. However, no exact upgrading/downgrading points are provided from the suggested default number of points. AutoCNV does not apply the scoring range setting and assigns the suggested default number of points for these scoring categories automatically. To enable user flexibility, a range is provided for most scoring categories in wAutoCNV (Fig. 2). An upgrading/downgrading point of 0.05 from the suggested default number of points was provided by wAutoCNV, which is consistent with the setting in the web-based ClinGen CNV Interpretation Calculator [1].

### Interpretation of 72 CNVs and 20 illustrative case examples using AutoCNV

To assess the performance of AutoCNV, we assessed 72 CNVs evaluated by external independent reviewers^2^ using AutoCNV. As a result, 100 % of CNVs were consistent with the reviewers’ classifications using AutoCNV, including 15 pathogenic, 3 likely pathogenic, 51 variants of uncertain significance (VUS), 1 likely benign and 2 benign.

Twenty illustrative case examples were also used to assess the performance of AutoCNV. As a result, 95 % of CNVs (19/20) were consistent with ClinGen verified clinical classification using AutoCNV (Supplementary Table 1), including 9 pathogenic, 4 VUS, 1 likely benign and 5 benign CNVs. The only difference was in Case X. The ClinGen verified “benign” duplication in Case X (Supplementary Table 1) was classified as “likely benign” by AutoCNV. We further investigated this difference in Case X. AutoCNV applied evidence 1 A (0), 3 A (0), 4 N (-0.9) and 5 F (0) for classification (point value − 0.9, likely benign), while ClinGen included all these points as well as 4D (-0.3). ClinGen applied 4D (individual case evidence-phenotype inconsistent) because numerous instances with varied indications were observed in their internal database. Therefore, a suggested point value of -1.2 and classification of “benign” was assigned, resulting in the inconsistent classification of the duplication in Case X.

AutoCNV can significantly reduce the annotation and classification burden by automating steps that can be automated. We compared the time spent by AutoCNV and complete manual interpretation for the 72 CNVs and 20 illustrative case examples for the results that can be automatically generated by AutoCNV. For human curation, more than 215 s were required for one CNV by manual interpretation. For automated generated pre-classification, AutoCNV only required an average of less than 5 milliseconds to obtain the result for one CNV, which was approximately 43,000 times faster than complete manual interpretation. The most time-consuming process for complete manual interpretation is database searching and results gathering.

### Interpretation of 64 CNVs from the ClinVar database

We further assessed the performance of AutoCNV using 64 CNVs from the ClinVar database (accessed on April 4th, 2021). The 64 CNVs consist of 28 pathogenic, 3 likely pathogenic, 32 VUS and 1 benign CNVs. As a result, 61 % (39/64) of the 64 CNVs were consistent with the original classifications when using AutoCNV (Supplementary Table 2).

28 % of the dis-concordant CNV classifications (7/25) between the original classifications in the ClinVar database and classifications using AutoCNV happened in the automating steps. The automated generated pre-classification of these 7 CNVs was classified as pathogenic or likely pathogenic by AutoCNV, while the original classification was VUS. One possible explanation is that some of the original classifications in ClinVar may be incorrect based on the 2019 ACMG/ClinGen Technical Standards. In addition, original classifications of “pathogenic” and “likely pathogenic” were classified as “VUS” in 15 CNVs and 2 CNVs by AutoCNV, respectively (Supplementary Table 2). According to the 2019 ACMG/ClinGen Technical Standards, evidences using cases from published literature, public databases, and/or internal lab data (Sec. 4) can not warrant the upgrade in these CNVs. With an original classification of “benign”, the duplication in RCV001270910 (proven in tandem) was classified as “VUS” by AutoCNV. According to the 2019 ACMG/ClinGen Technical Standards, no evidences from published literature (Sec. 4) support that this is a benign CNV.

### Interpretation of CNVs in the dbVar database

**Fig. 3:**
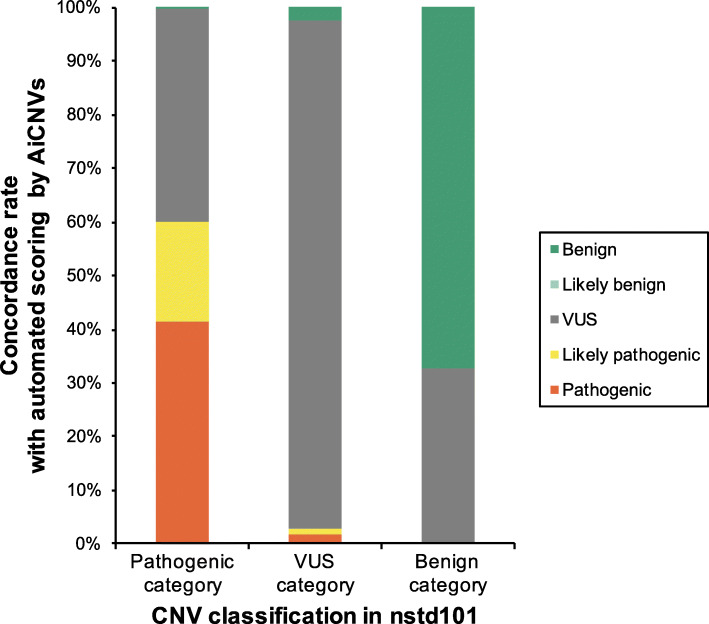
Automated interpretation of CNVs in nstd101 by AutoCNV

To apply AutoCNV in a larger database, we assessed 3,982 CNVs in study nstd101 from dbVar using AutoCNV, including 2,125 copy number losses (53 %) and 1857 copy number gains (47 %). These CNVs were classified into 3 categories (pathogenic category: pathogenic or likely pathogenic; VUS category; benign category: benign or likely benign) (Fig. 3). The CNV classifications by AutoCNV were based on the results that can be automatically generated by AutoCNV.

As a result, AutoCNV generated concordance rates of 56.3 % (1274/2263), 97.6 % (1202/1231) and 67.0 % (327/488) for the pathogenic category, VUS category and benign category, respectively. As shown in Fig. 3, most of the discordance occurred in pathogenic and benign categories, where pathogenic/likely pathogenic or benign/likely benign CNVs were classified as VUS by AutoCNV. One explanation for these findings is that the CNV classifications by AutoCNV were only based on the results that can be automatically analyzed. Manual adjustment step was needed before obtaining the final CNV classification. However, detailed evidences used for CNV classification in dbVar database are not available for this dataset. Another possible explanation is that is that some of the classification in nstd101 may be incorrect or incomplete. Part of the CNVs in nstd101 were collected from ClinVar. It has been reported that re-evaluation of 246 CNVs in the ClinVar database showed updated clinical classifications in more than 64 % of cases [13]. Discrepancies in assigning CNVs to either pathogenic or benign categories warrant further manual evaluation.

Notably, one benign CNV (nssv578203, copy number loss, chr11: 55,213,165 − 56,882,257) in nstd101 was classified as “likely pathogenic” by AutoCNV. This deletion contains more than 35 genes (49 genes), and a suggested point value of 0.9 and classification of likely pathogenic should be assigned (category 3 C). Further manual analysis showed that this deletion was submitted in 2012, and no detailed information was provided for the assessment of the deletion. This deletion might be incorrectly cataloged as benign in nstd101.

Two pathogenic CNVs (nssv579666 and nssv577383) in nstd101 were classified as “benign” by AutoCNV. nssv579666 is entirely within a common population variation (gssvL6983, chr1:196,757,278–196,796,716), which was deposited in the DGV database with a frequency of 12.45 % (case number > 1,000). According to this evidence, this deletion was classified as “benign” by AutoCNV (4O). Further manual interpretation showed no additional evidence in support of pathogenicity. nssv577383 is also entirely within a common population variation (gssvL23855, chr12:27,170,449–27,973,050), which was deposited in the DGV database with a frequency of 4 % (case number > 1,000). According to this evidence, this deletion was also classified as “benign” by AutoCNV (4O). nssv579666 and nssv577383 were submitted to nstd101 in 2012 and 2011, and no detailed information was provided for the assessment of these two deletions. Therefore, both deletions might be incorrectly cataloged as pathogenic in nstd101.

## Discussion

In this study, we developed a semiautomatic tool, AutoCNV, for CNV interpretation based on the standards. ClinGen has provided a tool (ClinGen CNV Interpretation Calculator) to evaluate the clinical significance of both copy number loss and copy number gain. Briefly, this tool is designed only to track and tally the points that have been assigned based on the observed evidence. AutoCNV, on the other hand, provides a complete package by providing both annotation information and scoring to facilitate the interpretation process. To date, AutoCNV has combined a total of 6 databases and provides a standardized pipeline for CNV interpretation. The main functionality of AutoCNV is to provide consistent and reliable CNV interpretation based on the standards. AutoCNV may help to enable reproducible CNV interpretation by the same clinical scientist and to facilitate consistent and reliable CNV interpretation by clinical scientists from the same or different labs.

For the purpose of automated interpretation, some criteria in the standards need to be clarified. To find a solution, these criteria were also discussed with the ACMG Document Support Team through e-mail. First, Table 2 (CNV interpretation scoring metric: copy-number gain) in the standards^2^ lists suggested points for CNVs overlapping with established TS genes (category 2A, 2B), HI genes (2H, 2I), and gene(s) of no established clinical significance (2L). However, there are no suggested points for CNVs that overlap with other disease-associated genes, such as the FGFR2 gene. As a solution, if a gene has not been evaluated by ClinGen Dosage Sensitivity, a suggested point value of 0 is assigned to the CNV by AutoCNV. The user can utilize Sec. 4 (evidence from the literature) to accumulate an appropriate number of points. Second, functionally important elements are used for initial assessment of genomic content in Sec. 1; however, no exact definition or database is available to determine functionally important elements. Regions with a haploinsufficiency/triplosensitivity score of 1, 2 or 3 in ClinGen are recognized as functionally important elements. This practice was also applied in the illustrative case examples from the standards [1]. Third, Table 2 in the standards^2^ listed suggested points values for CNVs overlapping with established benign copy-number gain genes or genomic regions (2 C-2G). However, there are two more scenarios (Supplementary Fig. 1): 1) the CNV overlaps but does not include additional protein-encoding genes while potentially interrupting protein-encoding genes (2E’), and 2) the CNV overlaps but does not include additional protein-encoding genes while not potentially interrupting protein-coding genes (2 F’). As a solution, CNVs with the scenario 2E’ is classified utilizing category 2E of the scoring metrics, while CNVs with the scenario 2 F’ is classified utilizing category 2 F of the scoring metrics by AutoCNV.

There are some limitations to the current version of AutoCNV. To assess the performance of AutoCNV compared to complete manual interpretation, we used 72 CNVs evaluated by external independent reviewers and 20 illustrative case examples for comparison. We do know that these CNVs are not enough to draw the conclusion that interpretation using AutoCNV is consistent with complete manual interpretation by experienced clinical scientists. Further assessment is still warranted.

In summary, we developed a highly automated CNV interpretation tool, AutoCNV, and a web-based server wAutoCNV (https://phoenix.bgi.com/autocnv/) for CNV interpretation based on the standards. AutoCNV combines a total of 6 databases and provides a standardized pipeline for CNV interpretation. Clinical scientists may use AutoCNV to accelerate and facilitate the annotation and interpretation process of CNVs, to improve the consistency and reliability of CNV interpretation and to conduct in-depth research and clinical diagnosis for CNV interpretation. The source code of AutoCNV is available on GitHub (https://github.com/zhonghua-wang/autocnv).

## Methods

### Annotation of CNV

AutoCNV enables users to input a genomic coordinate (start/end) of a given CNV, which is in the format of chromosome number: genomic coordinates (for example, chr1:1-1000). After the input of the genomic coordinate of a given CNV, annotation is conducted by AutoCNV to obtain genomic content, overlap information and gene number of the CNV (Fig. 1). The hg19/GRCh37 assembly of the human genome was used for annotation. In this process, a total of 6 databases were included in AutoCNV for annotation. RefGene (accessed on July 7th, 2020), ClinGen (accessed on July 7th, 2020), ClinVar (accessed on July 7th, 2020), GnomAD (V2.1.1) and DECIPHER (version 9.31) were employed to annotate genomic content, to establish/predict haploinsufficiency (HI) and triplosensitivity (TS) and to establish benign genes/genomic regions and gene number of the CNV. GnomAD (V2.1.1) and DGV (version DGV.GS.2016) were employed to annotate polymorphisms. For genes with multiple transcripts, the transcript selection criteria in AutoCNV are consistent with autoPVS1 [14], which is a classification tool for PVS1 interpretation. All the databases can be updated every two weeks automatically through our internal script.

### Scoring system of AutoCNV

For the 40 criteria for copy number loss, AutoCNV can automatically generate predictions on 18 criteria (45 %) to facilitate the process of interpretation. The remaining 22 criteria require the input of point values by users to generate final classification of the CNV according to case information from published studies, public databases, internal lab data, or inheritance pattern/family history for the sample being studied. For the 40 criteria for copy number gain, AutoCNV can automatically generate predictions on 16 criteria (40 %) to facilitate the process of interpretation. The remaining 24 criteria require input of point values to generate the final classification of the CNV according to case information from published literature, public databases, internal lab data, or inheritance pattern/family history for the patient being studied. In general, the framework of the scoring system of AutoCNV assigns points in ascending order (from Secs. 1 to Sec. 5). A given CNV goes through all the sections in the standards before obtaining the final point and CNV classification (Supplementary Fig. 2, Supplementary Fig. 3).

#### Scoring for Sec. 1: initial assessment of genomic content

For both copy number loss and gain, the scoring system of AutoCNV began from the automated scoring for the first evidence category (Sec. 1) of the CNV interpretation scoring metric (Supplementary Fig. 2, Supplementary Fig. 3) [1]. AutoCNV implements the database of RefGen (accessed on July 7th, 2020) and ClinGen (accessed on July 7th, 2020) for genomic content annotation (protein-encoding or functionally important elements). In this instance, AutoCNV uses human protein-coding genes (labeled with “protein-coding” in the RefSeq database) in the RefGene database (accessed on July 7th, 2020) for genomic content annotation of protein-encoding genes. Regions in ClinGen with a haploinsufficiency/triplosensitivity score of 1, 2 or 3 were employed to determine the functionally important elements. AutoCNV automatically assesses the genomic content of the CNV. For both copy number loss and gain, a suggested point of -0.60 is assigned to the CNV by AutoCNV if it does not contain protein-encoding genes or other known functionally important elements. A suggested point of 0 is assigned if the CNV contains protein-encoding or other known functionally important elements. Then, the CNV is further assessed for Sec. 2 of the evidence scoring metric.

#### Scoring for Sec. 2 (copy-number loss): haploinsufficiency (HI) or established benign evaluation

In this section, a given deletion is automatically evaluated for Sec. 2 of the CNV interpretation scoring metric by AutoCNV (Supplementary Fig. 2). In this evidence category, AutoCNV automatically evaluates the overlap of a deletion with established dosage-sensitive genes or genomic regions or any established benign genes or genomic regions. For the scoring of copy number loss, AutoCNV implements the ClinGen database (accessed on July 7th, 2020) for the annotation of HI or benign genes/genomic regions. To enable user flexibility, AutoCNV provides a range for most scoring categories when considering evidence of different relative strengths.

First, AutoCNV automatically assesses the overlap of the deletion with established HI genes/regions according to the annotation results.

If the given deletion completely spans one or more established HI genes or genomic regions (category 2 A), a suggested point of 1 is assigned. If a deletion partly overlaps the region of an established HI genomic region that contains no known HI gene(s) or if the deletion does not contain any HI genes in the HI genomic region (category 2B), 0 is assigned.

For deletions partly overlapping an established HI gene, further evaluation is conducted by AutoCNV according to the genomic content of the deletion. To determine the potential functional effect, AutoCNV considers both breakpoint location and involvement of coding sequence. When the deletion under evaluation partially overlaps with the 5’ end of the gene, a default point of 0.9 (range: 0.45-1) or 0 (range: 0-0.45) is assigned to the deletion when it involves (category 2 C-1) or does not involve (category 2 C-2) an additional coding sequence. When the deletion under evaluation partially overlaps with the 3’ end of the gene, the involved exons are further evaluated. If the deletion involves only the last exon, a default point of 0.9 (range: 0.45–0.9) is assigned to the deletion by AutoCNV when established pathogenic variants have been documented in that exon (category 2D-2). Here, established pathogenic variants refers to recorded pathogenic or likely pathogenic in ClinVar with an allele frequency < 1 % in GnomAD. Information of star level and time since last evaluation in ClinVar was not used. Otherwise (category 2D-3), a default point of 0.3 (range: 0-0.45) is assigned to the deletion by AutoCNV. If the deletion involves other exons in addition to the last exon (category 2D-4), a default point of 0.9 (range: 0.45–0.9) is assigned to the deletion by AutoCNV. When the deletion under evaluation is completely within an established HI gene (category 2E), the deletion effect on the protein is further evaluated by autoPVS1 [14], which is an automatic classification tool for supporting PVS1 interpretation under the guidance for interpreting loss-of-function (LoF) PVS1 ACMG/AMP variants [15]. In summary, PVS1 interpretation for exonic deletion is based on its effect on reading frame and NMD, importance of altered regions, high-frequency putative LoF variants and biologically relevant transcripts. If the truncated/altered region disrupts the reading frame and is predicted to undergo nonsense-mediated mRNA decay (NMD) and if the exon is present in biologically relevant transcripts, PVS1 can be applied, and a default point of 0.9 (range: 0.45–0.9) is assigned to the deletion by AutoCNV. If the truncated/altered region does not disrupt the reading frame or is not predicted to undergo NMD, the frequency of LoF variants in the altered region and the amount of protein removed by the deletion is further evaluated by autoPVS1. If the altered region is critical to protein function, PVS1_Strong is applied, and a default point of 0.45 (range: 0.3–0.9) is assigned to the deletion. If the protein function of the altered region is unknown, LoF variants in the region are not frequent in the general population, and exons are present in biologically relevant transcripts, PVS1_Strong or PVS1_Moderate is applied when the amount of protein removed by the deletion is > 10 % or < 10 %. Next, a corresponding default point of 0.45 (range: 0.3–0.9) or 0.3 (range: 0.15–0.45) is assigned to the deletion. If the deletion meets the conditions listed above, all evidence remains, but only the highest score is calculated.

Second, if no established HI gene/genomic region overlaps with the deletion, predicted HI genes are further analyzed. If it is shown that the deletion involves at least one gene with a GnomAD pLI score > = 0.9 (with the upper bound of the observed/expected confidence interval < 0.35) and a DECIPHER HI index of < = 10 % (category 2 H), a suggested point of 0.15 is assigned, and Sec. 2 is further analyzed for this deletion.

Finally, the established benign genes or genomic regions are analyzed. If the deletion is completely within established benign genes or genomic regions or the protein-encoding genes of the deletion are entirely the same as established benign genomic regions (category 2 F), a final point of -1 is then automatically calculated, and a final CNV classification of “benign” is arrived at and assigned for the deletion, then no further assessment is performed. If the deletion overlaps with an established benign CNV (but includes additional genomic material (category 2G)) or no benign CNVs overlap, then further assessment is conducted.

#### Scoring for Sec. 2 (copy-number gain): haploinsufficiency (HI), triplosensitivity (TS) or established benign evaluation

In this section, a given duplication is automatically evaluated for Sec. 2 of the CNV interpretation scoring metric by AutoCNV (Supplementary Fig. 3). In this evidence category, AutoCNV automatically evaluates the overlap of a duplication with established dosage-sensitive genes or genomic regions or any established benign genes or genomic regions. For the scoring of copy number gain, AutoCNV implements ClinGen (accessed on July 7th, 2020) for the annotation of TS genes/genomic regions, HI genes or established benign regions. To enable user flexibility, AutoCNV also provides a range in this category.

First, AutoCNV automatically assesses the overlap of the duplication with TS genes/regions according to the annotation results. If the given duplication completely spans one or more established TS gene or genomic region, a suggested point of 1 is assigned to the duplication.

Second, for duplications partly overlapping with the region of an established benign gene or genomic region, if the duplication is the same as a benign copy number gain (category 2 C), -1 is assigned. If the duplication involves additional genomic material (category 2G) or potentially interrupts protein-encoding genes (category 2E), 0 is assigned. Otherwise (category 2D, 2 F), a point of -1 is then automatically calculated. For category 2 F, a range score of (-1-0) is provided to users.

Finally, if the duplication is completely within an established HI gene (category 2I), autoPVS1 is also employed for assessment. PVS1 interpretation for exonic duplication is based on tandem status and effect on reading frame and NMD. If a duplication with reading frame disrupted and NMD predicted to occur is detected in tandem, PVS1 is applied, and a point of 0.9 is assigned to the duplication. If the duplication is assumed in tandem, PVS1_Strong is applied, and a point of 0.45 is assigned. For duplications partly overlapping the region of an established HI gene, categories 2 J and 2 K is further manually evaluated by the user according to the phenotype of the case. For duplications overlapping gene(s) of no established clinical significance (2 L), 0 is assigned.

#### Scoring for Sec. 3: gene number evaluation

As shown in Supplementary Fig. 2 and Supplementary Fig. 3, a given CNV is automatically evaluated and scored for protein-encoding gene number by AutoCNV. AutoCNV implements the RefGene database (accessed on July 7th, 2020) to annotate protein-encoding genes. After the input of the genomic coordinate of a given CNV, all the protein-encoding genes located in the genomic coordinate of a given CNV (with > = 1 bp overlap) will be recorded in the annotation step. In this step for gene number evaluation, AutoCNV will count the exact gene number for a given CNV. Then, for copy number loss, a suggested point of 0.45 is assigned automatically by AutoCNV to deletions containing between 25 and 34 protein-encoding genes (category 3B), and a suggested point value of 0.9 is assigned automatically to deletions containing more than 35 protein-encoding genes (category 3 C). For copy number gain, a suggested point value of 0.45 is assigned automatically to duplications containing between 35 and 49 protein-encoding genes (category 3B), and a suggested point value of 0.9 is assigned automatically to deletions containing more than 50 protein-encoding genes (category 3 C).

#### Scoring for Sec. 4: genomic content evaluation using cases from published literature, public databases, and/or internal lab data

For both deletions and duplications, manual evaluation of genomic content using cases from published studies and public databases is implemented in AutoCNV (Fig. 1). According to the scoring metric (4 A-4 N) in the standards, users can adjust this criterion according to individual case evidence in the manual adjustment step.

It is recommended that if a given CNV overlaps with a common population variation, category 4O can be applied [1]. In AutoCNV, common population variation is defined as when the frequency of the variant is > = 1 % (test samples > = 1,000) in the DGV standard dataset [16] or when the allele frequency is > = 1 % (total allele count > = 2,000) in the GnomAD databases [16]. If a given CNV is completely within a common variation with the same dosage (copy number loss or gain), a default score of -1 is assigned automatically by AutoCNV (category 4O). If a given CNV overlaps a common variation with the same dosage but does not contain other protein-encoding genes, the percentage of the overlap region is further calculated. If the percentage of the overlap region in the given CNV is > = 50 %, a default score of -1 is applied (category 4O). Otherwise, 4O cannot be used.

#### Scoring for Sec. 5: inheritance pattern/family history evaluation for patient being studied

Manual evaluation of inheritance patterns/family history for the case being studied is implemented in AutoCNV (Fig. 1). According to the scoring metric in the standards, users can adjust this criterion according to the family history of the case (if possible) in the manual adjustment step.

### Reporting: final scoring and CNV classification

According to the scoring metric of the standards, the scoring system of AutoCNV evaluates evidence and assigns points to a given CNV in ascending order (from Secs. 1 to Sec. 5). Finally, AutoCNV automatically calculates the final point value of a given CNV, and the corresponding CNV classification is also generated simultaneously (Fig. 1). AutoCNV enables users to apply points for individual evidence categories for a given CNV.

### Comparative analysis

To assess the performance of AutoCNV, 72 CNVs evaluated by external independent reviewers, 20 illustrative case examples [1] and 64 CNVs from the ClinVar database were used in this study. In the standards, 114 CNVs were used for validation of the CNV scoring metrics by outside reviewers and committee members [1]. Seventy-two CNVs (72/114) were evaluated by 2 independent reviewers, and both reviewers’ classifications matched using the CNV scoring metrics. The 20 illustrative case examples consist of 5 copy number losses and 15 copy number gains, including 4 case examples from the standards^2^ and 16 additional case examples (accessed on July 1st, 2020) from the CNV Technical Standards Web Series (https://clinicalgenome.org/tools/cnv-webinar/examples/). The 64 CNVs from the ClinVar database were more recent CNV submissions (submitted between November 2019 to April 2021). All the 64 CNVs were germline CNVs (containing more than 2 genes and with a CNV size of > 50 bp), including 34 copy number losses and 30 copy number gains. Comparative analysis of these CNVs (final classification) by complete manual interpretation and interpretation using AutoCNV (both of the automated interpretation step and manual adjustment step) were conducted simultaneously. All the methods were performed in accordance with the relevant guidelines and regulations.

## Supplementary Information

**Additional file 1.**

**Additional file 2.**

**Additional file 3.**

**Additional file 4.**

**Additional file 5.**

## Data Availability

Data sharing is not applicable to this article as no new data were created or analyzed in this study. The source code of AutoCNV is available on GitHub with a free license for noncommercial use (https://github.com/zhonghua-wang/autocnv). RefGene: http://www.ncbi.nlm.nih.gov/refseq. DECIPHER: https://decipher.sanger.ac.uk/. ClinGen: https://clinicalgenome.org/working-groups/dosage-sensitivity-curation/. DGV: http://dgv.tcag.ca/dgv/app/home. GnomAD: https://gnomad.broadinstitute.org/. ClinVar: https://www.ncbi.nlm.nih.gov/clinvar/.
