# Supplementary material for: AutoCNV: a semiautomatic CNV interpretation system based on the 2019 ACMG/ClinGen Technical Standards for CNVs
Source: BMC Genomics. 2021 Oct 6;22:721. doi: 10.1186/s12864-021-08011-4 (PMC8496072; doi:10.1186/s12864-021-08011-4)
Supplement: Supplementary file 1 — Additional file 1. [file 12864_2021_8011_MOESM1_ESM.pdf]

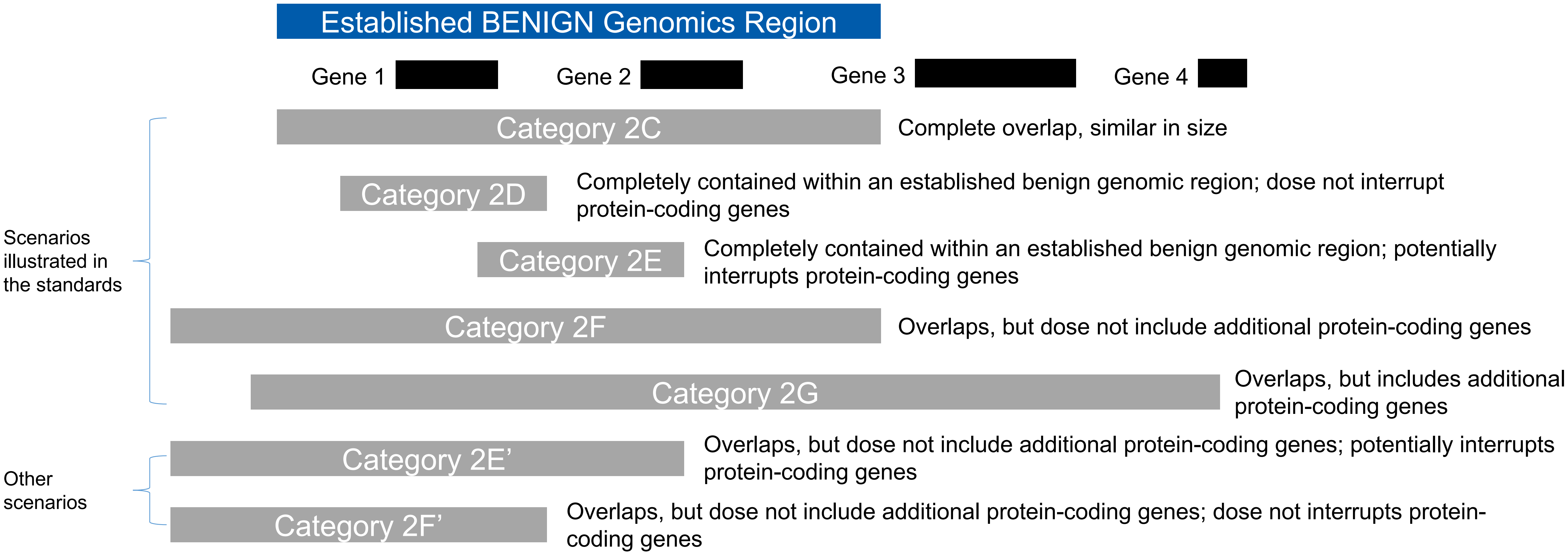
Established BENIGN Genomics Region
Gene 1
Gene 2
Gene 3
Gene 4
Scenarios illustrated in the standards
Category 2C
Complete overlap, similar in size
Category 2D
Completely contained within an established benign genomic region; dose not interrupt protein-coding genes
Category 2E
Completely contained within an established benign genomic region; potentially interrupts protein-coding genes
Category 2F
Overlaps, but dose not include additional protein-coding genes
Category 2G
Overlaps, but includes additional protein-coding genes
Other scenarios
Category 2E’
Overlaps, but dose not include additional protein-coding genes; potentially interrupts protein-coding genes
Category 2F’
Overlaps, but dose not include additional protein-coding genes; dose not interrupts protein-coding genes
