# Supplementary figures and images for: AutoCNV: a semiautomatic CNV interpretation system based on the 2019 ACMG/ClinGen Technical Standards for CNVs
Source: BMC Genomics. 2021 Oct 6;22:721. doi: 10.1186/s12864-021-08011-4 (PMC8496072; doi:10.1186/s12864-021-08011-4)

## Section 1

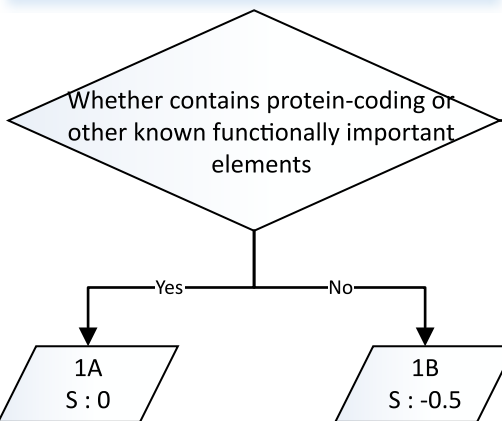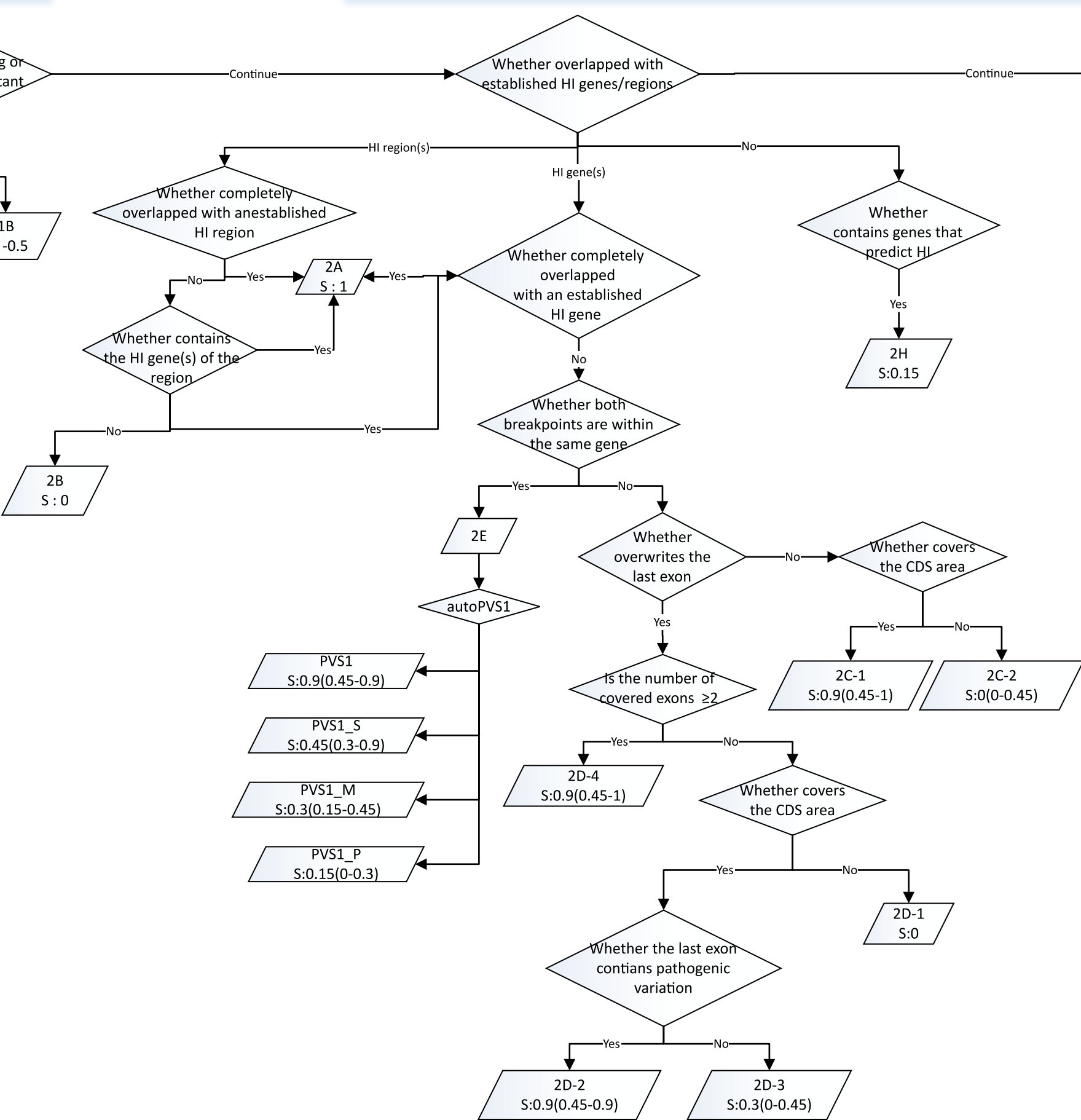

## Section 3

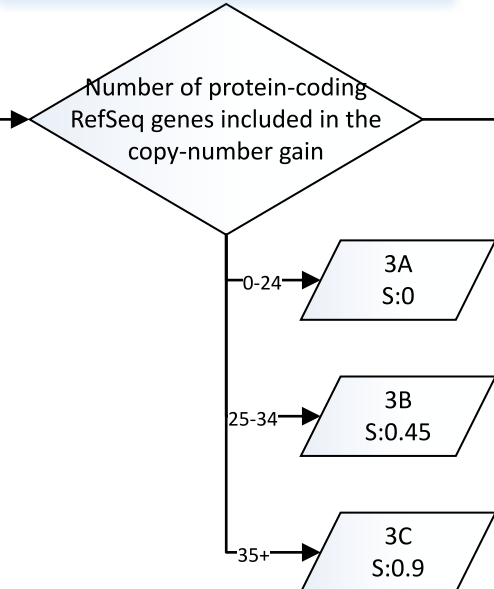

## Section 4

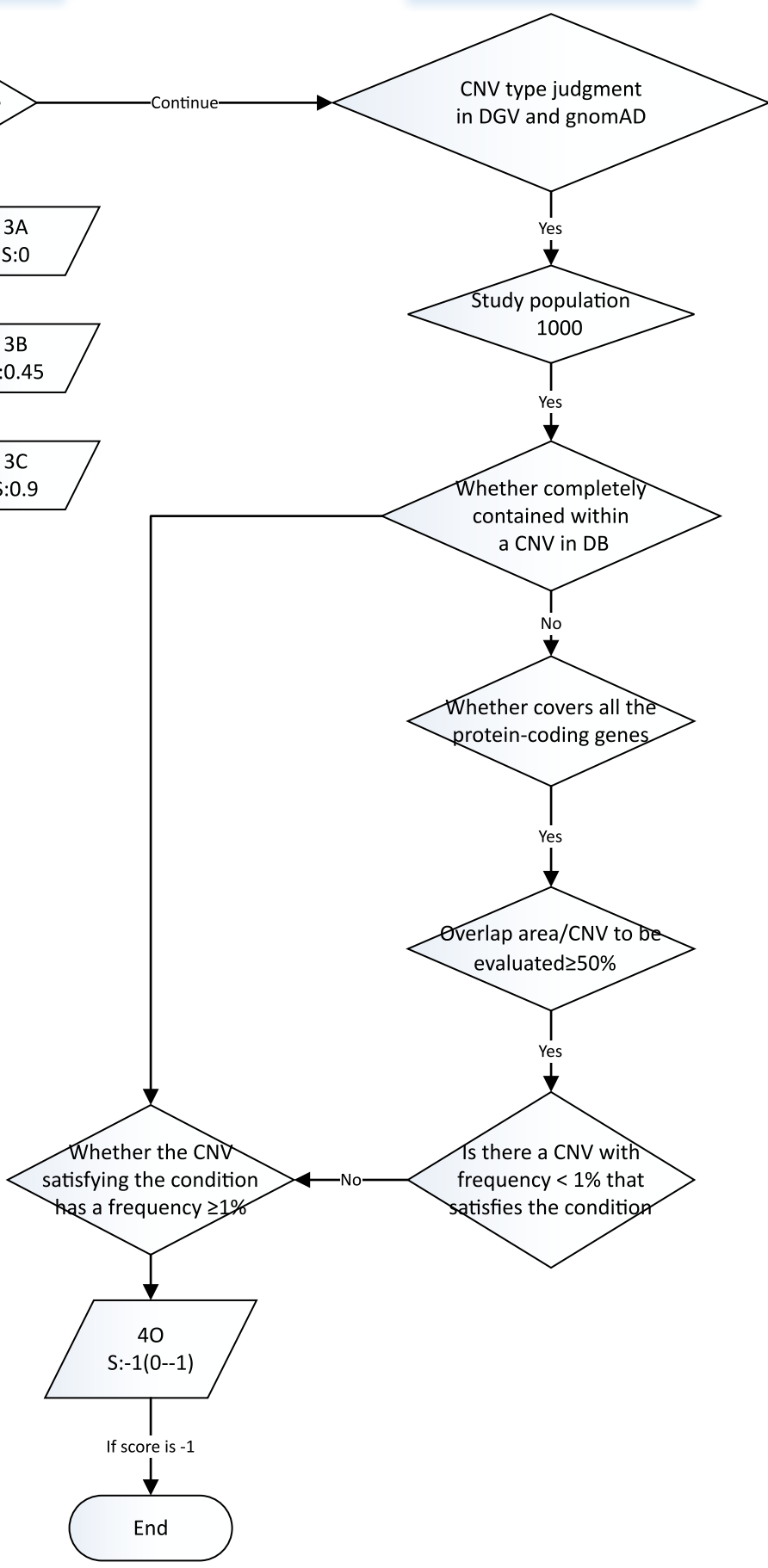

Supplement: Supplementary file 2 — Additional file 2. [file 12864_2021_8011_MOESM2_ESM.pdf]

## Automated scoring for copy-number gain

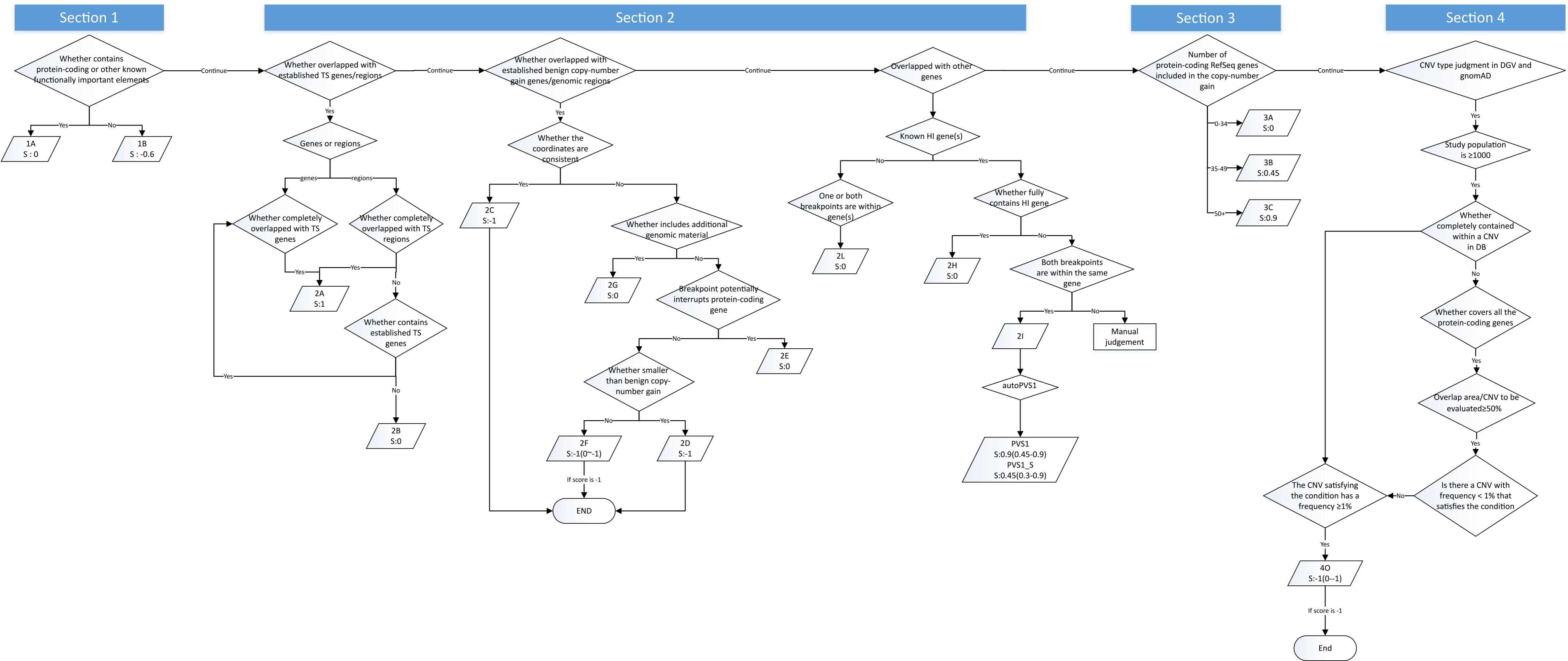

Supplement: Supplementary file 3 — Additional file 3. [file 12864_2021_8011_MOESM3_ESM.pdf]
