# Supplementary material for: AutoCNV: a semiautomatic CNV interpretation system based on the 2019 ACMG/ClinGen Technical Standards for CNVs
Source: BMC Genomics. 2021 Oct 6;22:721. doi: 10.1186/s12864-021-08011-4 (PMC8496072; doi:10.1186/s12864-021-08011-4)
Supplement: Supplementary file 4 — Additional file 4. [file 12864_2021_8011_MOESM4_ESM.pdf]

Table S1 Comparative CNV interpretation between AutoCNV and complete manual interpretation in twenty illustrative case examples

| Patient | Chromosome | Start | End | Type | Original Classification | Classification using AutoCNV |
|---|---|---|---|---|---|---|
| Case 2 | chr17 | 41784108 | 42438203 | Duplication | VUS | VUS |
| Case 4 | chr22 | 18912231 | 21465672 | Duplication | Pathogenic | Pathogenic |
| Case W | chr5 | 125989631 | 126295396 | Duplication | Pathogenic | Pathogenic |
| Case X | chr2 | 45408934 | 45976420 | Duplication | Benign | Likely benign |
| Case Z | chr19 | 18291753 | 18311626 | Duplication | Benign | Benign |
| Case 1 | chr12 | 27715516 | 29628080 | Deletion | VUS | VUS |
| Case 3 | chr19 | 43242796 | 43741310 | Deletion | Benign | Benign |
| Case U | chr9 | 108597937 | 111269478 | Deletion | Pathogenic | Pathogenic |
| Case V | chrX | 23223505 | 23660309 | Deletion | Pathogenic | Pathogenic |
| Case Y | chr4 | 69373811 | 69491113 | Deletion | Benign | Benign |
| Case A | chr12 | 12864101 | 14983330 | Deletion | Pathogenic | Pathogenic |
| Case B | chr11 | 45904399 | 46480747 | Deletion | Pathogenic | Pathogenic |
| Case C | chr18 | 53049652 | 53134356 | Deletion | Pathogenic | Pathogenic |
| Case D | chr22 | 40654201 | 40659533 | Deletion | VUS | VUS |
| Case E | chr15 | 30507853 | 30807921 | Deletion | Benign | Benign |
| Case F | chr1 | 1379519 | 1435227 | Deletion | Likely Benign | Likely benign |
| Case G | chr13 | 20812000 | 21012000 | Deletion | Benign | Benign |
| Case H | chr17 | 553250 | 1353250 | Deletion | Pathogenic | Pathogenic |
| Case I | chr22 | 31645547 | 32807482 | Deletion | Pathogenic | Pathogenic |
| Case J | chr3 | 190380498 | 191783134 | Deletion | VUS | VUS |
