# Supplementary material for: AutoCNV: a semiautomatic CNV interpretation system based on the 2019 ACMG/ClinGen Technical Standards for CNVs
Source: BMC Genomics. 2021 Oct 6;22:721. doi: 10.1186/s12864-021-08011-4 (PMC8496072; doi:10.1186/s12864-021-08011-4)
Supplement: Supplementary file 5 — Additional file 5. [file 12864_2021_8011_MOESM5_ESM.pdf]

Supplementary Table 2 CNV interpretation using AutoCNV in 64 CNVs from the ClinVar database

RCVaccession	Chromosome	Start	End	Type	Original Classification	Classification using AutoCNV
RCV001293382	11	11835569	118373112	Deletion	P	P
RCV001293373	9	97834573	107199088	Deletion	P	P
RCV001260503	10	81585301	89101700	Deletion	P	P
RCV001374461	3	68939251	72700418	Deletion	P	P
RCV001293370	22	18886915	21463730	Deletion	P	P
RCV001293366	22	18889490	21463730	Deletion	P	P
RCV001314756	14	65937790	68354021	Duplication	VUS	VUS
RCV001301079	12	6945914	9027627	Duplication	VUS	VUS
RCV001295457	20	60831241	62664346	Duplication	VUS	VUS
RCV001172267	7	72657228	74160300	Deletion	P	P
RCV001293377	16	14968859	16363239	Deletion	P	P
RCV001293368	22	42356886	43684002	Deletion	P	P
RCV001293379	9	139764148	141066491	Deletion	P	P
RCV001260502	14	22006109	23241448	Deletion	VUS	VUS
RCV001032318	2	165946640	167168286	Duplication	VUS	VUS
RCV001031254	11	117856768	118972385	Duplication	VUS	VUS
RCV001304384	11	117856768	118972385	Duplication	VUS	VUS
RCV001346193	9	34458994	35072710	Duplication	VUS	VUS
RCV001345686	9	35683146	36277049	Duplication	VUS	VUS
RCV001172265	X	102632399	103221016	Deletion	P	P
RCV001271073	X	69637865	70220983	Duplication	VUS	VUS
RCV001293374	15	75596353	76103430	Deletion	P	P
RCV001031609	8	74888367	75279355	Duplication	VUS	VUS
RCV001364734	21	44838130	45196150	Duplication	VUS	VUS
RCV001305754	1	948954	1284445	Duplication	VUS	VUS
RCV001255130	3	12630535	12808483	Duplication	VUS	VUS
RCV001032472	X	53276131	53449569	Duplication	VUS	VUS
RCV000803281	19	55644273	55768277	Duplication	VUS	VUS
RCV001033047	18	29078205	29178648	Duplication	VUS	VUS
RCV001305882	20	61990880	62078210	Duplication	VUS	VUS
RCV000824661	21	35736455	35821942	Duplication	VUS	VUS
RCV001031126	14	23856728	23885051	Duplication	VUS	VUS
RCV001370406	14	23861761	23889453	Deletion	VUS	VUS
RCV001290038	X	154277426	154299480	Deletion	VUS	VUS
RCV001033362	16	2089925	2098066	Duplication	VUS	VUS
RCV001200600	16	79245609	79245697	Deletion	VUS	VUS
RCV001256153	15	90451636	90452607	Deletion	VUS	VUS
RCV001257570	17	60683462	60722398	Deletion	LP	VUS
RCV001257508	13	20802727	21034768	Deletion	P	VUS

RCV001319817	5	136633338	140998481	Duplication	VUS	LP
RCV001031775	17	6589506	8151374	Duplication	VUS	LP
RCV001316565	16	624055	2153916	Duplication	VUS	LP
RCV001305099	X	48382160	49856876	Duplication	VUS	LP
RCV000537451	20	61977556	62159505	Deletion	VUS	P
RCV001300221	1	130980840	248900000	Duplication	VUS	P
RCV001325176	15	32964879	91358519	Duplication	VUS	LP
RCV001256147	6	116734559	123648104	Deletion	P	VUS
RCV001290060	10	49033586	52417694	Deletion	LP	VUS
RCV001172266	4	83196931	85540706	Deletion	P	VUS
RCV001257507	13	20797127	21105945	Deletion	P	VUS
RCV001199824	22	21822774	23025613	Deletion	P	VUS
RCV001268958	17	54290100	54844894	Deletion	P	VUS
RCV001270921	13	95209609	95292265	Deletion	P	VUS
RCV001293383	14	86089631	88429727	Deletion	P	VUS
RCV001293375	22	23699269	24992266	Duplication	P	VUS
RCV001199823	5	176517797	176779857	Duplication	P	VUS
RCV001270910	X	30939526	31362638	Duplication	B	VUS
RCV000417215	16	204300	227000	Deletion	P	VUS
RCV001078233	16	213800	230200	Deletion	P	VUS
RCV001078235	16	221844	231553	Deletion	P	VUS
RCV001078236	16	221999	231399	Deletion	P	VUS
RCV001194694	2	228481316	228609111	Deletion	P	VUS
RCV001268961	X	153011909	153063825	Deletion	P	VUS
RCV001095544	9	96160235	97428496	Duplication	LP	VUS

P: pathogenic; LP: likely pathogenic; VUS: variants of uncertain significance; LB: likely benign; B: benign
